# Molecular mechanisms of obesity predisposes to atopic dermatitis

**DOI:** 10.3389/fimmu.2024.1473105

**Published:** 2024-11-04

**Authors:** Dajin Shang, Shengnan Zhao

**Affiliations:** ^1^ School of China Medical University, Shenyang, Liaoning, China; ^2^ Department of Dermatology, The First Hospital of China Medical University, Shenyang, China

**Keywords:** atopic dermatitis, obesity, immune, adipokines, cytokines

## Abstract

Obesity is a prevalent metabolic disease that reduces bacterial diversity, colonizes the epidermis with lipophilic bacteria, and increases intestinal pro-inflammatory species, all of which lead to impaired epithelial barriers. Adipose tissue secretes immunomodulatory molecules, such as adipokines, leptin, and adiponectin, which alters the morphology of adipocytes and macrophages as well as modulates T cell differentiation and peripheral Th2-dominated immune responses. Atopic dermatitis (AD) and obesity have similar pathological manifestations, including inflammation as well as insulin and leptin resistance. This review examines the major mechanisms between obesity and AD, which focus on the effect on skin and gut microbiota, immune responses mediated by the toll like receptor (TLR) signaling pathway, and changes in cytokine levels (TNF-a, IL-6, IL-4, and IL13). Moreover, we describe the potential effects of adipokines on AD and finally mechanisms by which PPAR-γ suppresses and regulates type 2 immunity.

## 1 Introduction

Atopic dermatitis (AD) is a chronic, recurrent, inflammatory, pruritic dermatosis with complex pathophysiology, involving disruption of the epidermal barrier, microbial dysbiosis within affected lesions, and Th2 immune responses to skin allergens (1).Impaired skin barrier function is thought to be both a cause and a consequence of AD. Severe atopic dermatitis has been linked to deficiencies in the filaggrin (FLG) protein or antimicrobial peptides (2). Increased inflammatory cell infiltration is observed in AD lesions, including T cells, dendritic cells, macrophages, mast cells, and eosinophils (3).These often precipitate increased cytokines including thymic stromal lymphopoietin (TSLP), interleukin-1 (IL-1), IL-4, IL-5, IL-6, IL-25, IL-33, and transforming growth factor-β (TGF-β), which promote inflammation and immune activation. Upregulated expression levels of IL-12, interferon-γ (IFN-γ), and granulocyte-macrophage colony-stimulating factor (GM-CSF) are detected during chronic phase (4). Additionally, Th17 and Th22 cell cytokines, such as IL13, IL-17, CCL17, tumor necrosis factor-α (TNF-α), and IL-22, promote the formation of chronic skin lesions in AD (4). Atopic sensitization is also associated with IL9, IL33, and IL33R expression during infancy. In addition, AD could be aggravated by dysbiosis or imbalances in microbial species diversity and environmental factors including diet (5).

Obesity, a major health problem, is a result of metabolic syndrome in adipose tissue and is linked to various chronic immune disorders (6). Studies have shown that adipose expansion in early and chronic obesity activates an inflammatory program, altering the immune phenotype to a pro-inflammatory status (7). Adipose depots usually release cytokines, chemokines, and adipokines that coordinately regulate other immune cells, including eosinophils, mast cells, and macrophages in an M2-polarized or alternately activated state (7). Treatment that reduces obesity symptoms could reduce epidermal thickness and eosinophil/mast cell infiltration, along with a reduction in IgE, IL-4, IL-6, TNF-α, and AD-like lesions (8).

Currently, the association of obesity/overweight with AD is not conclusive. Studies addressing obesity in infancy or early childhood (age < 2 years) and AD have found a positive association; from childhood into adulthood; but this was not observed in other cross-sectional studies (9). It has also been shown that obesity may have a pathogenic function in AD. Obese adults are more likely to develop moderate-to-severe AD (10). Investigations have revealed that higher levels of serum IgE and cutaneous mRNA levels of TNF-α, IL-13, and IL-31 are associated with more severe AD in rat models with a higher body weight than those with lower body weight (11). Moreover, IL-5 serum level correlated with both body mass index (BMI) and waist circumference (12). Collectively, these findings indicate that obesity may predispose individuals to or exacerbate AD. The discrepancies observed here may be explained by differences in study designs, the diagnostic criteria of AD, regional differences, and by the varied definitions of overweight and obesity used across studies. Although the potential mechanisms by which obesity contributes to AD are not fully understood, several potential mechanisms should be considered.

In obese or overweight individuals, low levels of adiponectin and PPAR-γ trigger a cascade of events. First, these low levels lead to increased production of cytokines and chemokines. These signaling molecules then activate macrophages and T cells, further promoting inflammation. The resulting inflammatory state can further elevate leptin levels, which can create a vicious cycle by further suppressing adiponectin and PPARγ (13). The leptin secreted in obese or overweight individuals can upregulate the expression levels of cytokines and chemokines, thereby increase the risk of AD. The increase in AD-related cytokines and chemokines, along with the decrease in PPAR-γ, not only induces inflammation in adipose tissue but also triggers insulin resistance and leptin resistance. This results in adipocyte hypertrophy, adipose tissue hyperplasia, and lipid accumulation, causing obesity (13). Imbalance in gut microbiota can increase an individual’s susceptibility to AD by disrupting mucosal immune tolerance. This disruption can affect skin homeostasis through its influence on the signaling pathways that maintain healthy skin barrier function. However, research suggests that changes in gut microbiota alone are likely not enough to trigger the development of AD. The interaction between specific microbial communities and the immune system, as well as other external factors such as diet, may explain the pathogenesis of AD (14).

## 2 Correlation between atopic dermatitis and obesity

Among the numerous research records, the results of various studies are not consistent, and the specific role of obesity in atopic dermatitis is not clear, and further exploration is needed. However, more and more evidence shows a certain correlation between atopic dermatitis and obesity. Obesity is one of the comorbidities of AD, and it has been confirmed as one of the risk factors for AD, which can also exacerbate the severity of AD. Compared to non-AD patients, AD patients have a higher probability of developing obesity, and the impact of obesity is more pronounced in the pediatric population. This implies that early-onset obesity in the childhood stage can increase the likelihood of developing AD (15). In a study of datasets in children, it was found that the prevalence of AD peaked early in the age group of 1 to 6 years, with a gradual downward trend (16). In a cohort study, AD was found to be associated with shorter stature, higher BMI, and lower weight in children during childhood, There was a clear association between AD and obesity in children before the age of 5,The association gradually weakened around the age of 5,As children grew, the association between AD and BMI was not consistent throughout childhood, further studies are needed to measure the long-term association and eliminate the impact of diet, sleep, etc. (17) Similarly, one study found that obesity was also significantly associated with the presence of AD in adults (18). Apart from age, geographical variations and gender differences may also influence the clinical presentation of AD and obesity. For instance, in North America and Asia, there is a correlation between increased prevalence of obesity and AD (19). In another study, it was found that there is a positive correlation between obesity and AD occurrence in the female population (20). The interaction between obesity and AD is mediated by various cytokines, immune mediators, and chemokines. Due to the interconnection of these two conditions, alleviating one may potentially prevent or mitigate the progression of the other disease, thereby effectively managing the conditions and enhancing individual health status (13).

## 3 The role of the microbiota in obesity and AD

Studies have reported a cross-talk mechanism between the skin and the gut. Dysbiosis in gut microbiota potentially disrupts microbial metabolites and pro-inflammatory factors as well as Th2 immune response, causing skin inflammation (21). Furthermore, infants with AD exhibit reduced levels of lactobacilli and bifidobacteria in their gut microbiota. There is also an increased proportion of Escherichia coli, Clostridium difficile, and Staphylococcus aureus (22). Obesity reduces gut microbial diversity and beneficial microbes in the gut, including Lactobacillus, and Bifidobacterium. A high-fat and high-sugar dietary habit decreases the abundance of beneficial bacteria including bifidobacteria and lactobacilli. This dietary pattern can also induce increased gut permeability and increased expression of inflammatory markers including TNF-α (23). The abundance of lactobacilli and bifidobacteria was decreased in obese mice models, causing higher circulating levels of LPS, promoting NF-kB pathway activation through TLR4 signaling pathway, thereby enhancing inflammatory response (24). Additionally, Corynebacterium colonization of the epidermis was associated with BMI. One study revealed that a high-fat diet increased skin Corynebacterium species and free fatty acids in mice (25). Gut microbiota analysis revealed reduced abundance of Ruminococcaceae in fecal samples of atopic eczema infants. Interestingly, the relative abundance of Ruminococcus was inversely related to TLR2-induced IL-6 and TNF-α in IgE-associated eczema (26). Ruminococcaceae was more abundant in the gut microbes of mice with normal weight than in obese mice (27). Moreover, the diversity of Bacteroidetes and Actinobacteria was reduced in atopic eczema infants compared to healthy controls (28). A new polysaccharide, ARS, has been shown to reverse or resist high-fat-diet-induced obesity. It appears to function by increasing the diversity of gut microbiota and optimizing the ratio of Firmicutes to Bacteroidetes (29). Topical treatments for AD, including corticosteroids, antibiotics, and calcineurin inhibitors, increase species diversity of the epidermis, including Streptococcus, Cutibacterium, and Corynebacterium spp (30). Functional foods that prevent obesity increase intestinal microbial diversity and beneficial bacteria (Bifidobacterium, Alloprevotella, and Lactobacillus) and, at the same time decrease harmful bacteria (Staphylococcus and Corynebacterium 1) (31). Research has shown that “plant-based foods” intake suppresses moderate-to-severe AD (32). L. plantarum LM1004 significantly improves the restoration of AD-like symptoms accompanied by decreased levels of Th2 and Th17 cell transcription factors, increased transcription factors of Treg and Th1 cells, and upregulated FLG expression (33). Gut inflammation and gut barrier leakage activate skin epithelial cells and promote the recruitment of T cells in patients with Omenn syndrome, thus exacerbating skin inflammation (34). The above findings revealed that BMI and diet influence the composition of skin microbiota and susceptibility of an individual to AD.

## 4 The role of the signaling pathways in obesity and AD

### 4.1 JAK-STAT pathway

The Janus kinase (JAK) - signal transducer and activator of transcription (STAT) pathway plays a crucial role in the immunological and physiological processes of AD and obesity. In the context of immune responses related to AD in the Th2 immune reaction, the binding of IL-4 and IL-4R stimulates the phosphorylation of JAK1 and JAK3, leading to the activation and phosphorylation of IL-4Rα and STAT6 (35).Furthermore, IL-4 and IL-13 can bind to the type II IL-4R, inducing the phosphorylation of JAK1 and TYK2, subsequently activating and phosphorylating STAT3 and STAT6. This results in the downregulation of FLG expression and impairment of skin barrier function, as well as an increase in the production of TSLP, IL-25, and IL-33 in keratinocytes (36). TSLP is a Th2 cell cytokine that can activate dendritic cells to drive Th2 cell differentiation and produce IL-4, IL-5, and IL-13 (37). Binding of the TSLPR heterodimeric receptor results in the interaction with JAK1 and JAK2, leading to phosphorylation and activation of STAT5 (38). IL-5 can also trigger the phosphorylation of JAK1 and JAK2 by binding to its corresponding receptor, resulting in the activation of STAT1, STAT3, and STAT5 (39). For Th17 immunity, the JAK-STAT pathway does not appear to be directly involved in Th17 signal transduction. However, research has shown that STAT3 is crucial for the proliferation and survival of Th17 cells (40). In Th1 immune responses, IL-12 signaling occurs through the binding of IL-12 to a heterodimeric receptor composed of IL-12Rβ1 and IL-12Rβ2 subunits (41). Subsequently, this interaction triggers the activation of JAK2 and TYK2, leading to the activation of STAT4-mediated signaling, and to a lesser extent, signaling mediated by STAT1, STAT3, and STAT5 (42). In skin function regulation, excessive activation of JAK1 can lead to overexpression of serine proteases in the skin, thereby impairing skin barrier function. Additionally, the STAT3 signaling molecule is a key transcription factor that regulates the differentiation of keratinocytes and maintains skin integrity (43). Secretory factors produced by adipocytes can participate in the JAK-STAT signaling pathway. In mice fed a high-fat diet, leptin secretion is associated with increased expression of STAT3 target genes. STAT3 can promote fat breakdown and inhibit fat synthesis (44). In the low-grade systemic inflammation associated with obesity, interferon-gamma (IFN-γ) secreted by CD4+ and CD8+ lymphocytes can activate STAT1 in adipocytes, leading to dysfunctional adipocyte function and insulin resistance (44). Studies have also shown that mice lacking STAT4 under high-fat diet feeding conditions can reduce adipose tissue inflammation, prevent insulin resistance, and improve glucose homeostasis (45). Additionally, STAT5 directly regulates the expression of key transcription factor PPARγ involved in adipocyte differentiation. STAT6 in macrophages is also essential for the browning of white adipose tissue (44). Inhibiting the JAK-STAT pathway pharmacologically can lead to downregulation of interferon response, resulting in the accumulation of UCP1 and browning of adipocytes (46).

### 4.2 TLR pathway

TLRs recognize and bind to pathogen-associated molecular patterns (PAMPs) including peptidoglycans, lipopolysaccharides, and yeast polysaccharides, initiating a cascade of signaling events. Research has found that the TLR2-mediated immune signaling pathway is impaired in patients with AD. Blocking the TLR2 pathway inhibits pro-inflammatory cytokines (IL-6, IL-8, and IL-1β) and promotes the expression of tight junction proteins, hence restoring the epidermal barrier in AD (47). On the other hand, TLR4 modulates the immune balance in AD. Its activation can impair Th1 immune response, exacerbate Th2 inflammatory response, induce migration of skin dendritic cells (DCs), and promote IL-22 expression in naive CD4 T cells, resulting in incremental proliferation of keratinocytes and inflammatory infiltration (48). TLR3 expression in primary sensory neurons potentially induces itch (49). Clinical studies have shown that TLR5 and TLR9 upregulation in umbilical cord blood may significantly reduce the incidence of AD. TLR signaling pathway influences AD development. A high level of LDL-C is also a risk factor for AD. Adolescents with AD have significantly higher total cholesterol and low-density lipoprotein cholesterol (LDL-C) levels than those without AD (50). Saturated fatty acids (SFAs) and free fatty acids (FFAs) increase upon high-fat intake. SFAs can promote inflammatory signaling in macrophages via the plasma membrane (51). Moreover, an increase in SFA levels promotes the synthesis of endocannabinoids, which can cause NLRP3 inflammasome activation in macrophages of diet-induced obese mice (52). NLRP3 inflammasome activation promotes the cleavage of pro-IL-1βand pro-IL-18, which stimulates the release of various active cytokines. IL-1β can stimulate the production of IL-6. A previous study found that IL-6 was increased in lesion of moderate to severe AD compared to normal skin (53). Obese patients often have increased serum uric acid levels and uric acid crystals which can act as NLRP3 activators, thereby releasing pro-inflammatory cytokines (IL-1β). Uric acid crystals can also activate the immune system by facilitating the production of reactive oxygen species (ROS) and activating the NF-kB and MAPK pathways (54). When deposited in adipose tissue and immune cells, cholesterol crystals can also activate the NLRP3 inflammasome through similar mechanisms mentioned above (55). NLRP3 inflammasome activation promotes pro-IL-1β and pro-IL-18 cleavage, hence releasing various active cytokines including IL-6. IL-6 is significantly upregulated in lesions of moderate to severe AD compared to normal skin (53).

## 5 The role of cytokines and adipokines in obesity and AD

Analysis of bodies of obese individuals has revealed the presence of metabolic abnormalities, oxidative stress, mitochondrial dysfunction, impaired immune function, and chronic low-grade inflammation (56). The infiltration of inflammatory cells into white adipose tissue (WAT) causes dysfunction of adipocytes and metabolic functions. White adipose tissue (WAT) comprises immune-regulatory cells, including M2-like adipose tissue macrophages, Tregs, Th2 cells, NK cells, and eosinophils. The quantity and phenotype of these cells vary between obese and lean individuals. Adipose secretes various cytokines, chemokines, and adipokines, which play a key role in regulating immune processes. Elevated lipid storage induce adipocyte hypertrophy, hypoxia, and cell death triggering the secretion of proinflammatory cytokines by adipocytes, including TNF-α, IL-6, IL-8, and MCP-1 (57). For lean individuals, regulatory and immunosuppressive cells promote the clearance of dead adipose tissue, suppressing adipose tissue cell proliferation, and secreting anti-inflammatory cytokines (IL-10, IL-4, IL-13, and IL-1Rα) (58).

### 5.1 TNF-α

TNF-α is a cytokine that plays a role in inflammation and immune responses (59). It can activate inflammatory responses, leading to the release of inflammatory cytokines and triggering inflammation. In patients with AD, TNF-α levels are typically elevated, and correlated with the severity of AD (60). High levels of TNF-α are associated with the inflammation and itching symptoms of AD. TNF-α can also promote the proliferation of skin keratinocytes and the synthesis of keratin, thereby promoting the onset of AD. The use of TNF-α inhibitors can help alleviate skin barrier dysfunction in AD patients and improve skin barrier function (61). Upregulated TNF-α in obesity activates the NF-κB pathway via a c-Jun N-terminal kinase-dependent pathway, resulting in downregulated expression of epidermal barrier proteins, including FLG and loricrin (LOR) (62). Elevated levels of TNF-α in obese individuals may disrupt the balance between Th1 and Th2 cells. TNF-α promotes Th1 immune response by enhancing the production of pro-inflammatory cytokines such as interferon-gamma (IFN-γ) and interleukin-2 (IL-2) (63). Obesity alters skin properties, increasing surface roughness, and decreasing water content thus causing significant redness accompanied by an increase in skin blood flow. Correlation analysis revealed a significant correlation between water content and TNF-α levels in the stratum corneum (64). Moreover, obesity changes the baseline levels of serum cytokines/adipocytokines IL-6, TNF-α, and CRP/hs-CRP (65). Research has shown higher serum TNF-α levels in obese individuals than in individuals with normal weight (65).This physiological activity may be associated with the action of leptin produced during obesity (66). Leptin can augment the secretion of inflammatory cytokines including TNF-α, resulting in an inflammatory environment (67). Simultaneously, exposure to an inflammatory environment can increase leptin expression in adipose tissue, creating a feedback loop that promotes inflammation (68). TNF-α secreted by M1 pro-inflammatory adipose tissue macrophages initiates a cascade reaction by activating the NF-κB and JNK pathways. TNF-α may stimulate the production of pro-inflammatory cytokines including IL-6, but reduce anti-inflammatory cytokine levels, including adiponectin in the inflammatory response (69). Adiponectin inhibits the TNF-α and IL-6 production in macrophages at the same time increasing levels of anti-inflammatory mediators (IL-10 and IL-1 receptor antagonist) (70, 71). Adiponectin-deficient mice exhibit an increased number of M1 macrophages in their adipose tissue, further improving cytokine production including TNF-α, IL-6, and MCP-1 (72). Additionally, TNF-α can cause the generation of reactive oxygen species (ROS) by binding to specific receptors (73).

### 5.2 IL-6

Research on the relationship between IL-6 levels in obesity and AD is limited.IL-6 regulates autoimmune and chronic inflammatory diseases. IL-6 signaling exerts pleiotropic effects via two primary pathways, the classical signaling and trans-signaling pathways. In the classical signaling pathway, IL-6 binds to IL-6R on the cell membrane, followed by interaction with membrane-bound gp130, hence activating JAK to initiate intracellular signaling (74). In the trans-signaling pathway, the extracellular portion of IL-6R can be proteolytically cleaved to form soluble sIL-6R. IL-6 subsequently binds to sIL-6R and continues to bind to gp130, initiating intracellular signaling (74). Activation of the classic IL-6 signaling pathway promotes macrophages polarized to M2 phenotype via upregulated IL4 response despite causing proinflammatory actions of T cells (75). Serum IL-6 levels in AD patients are significantly higher than that in healthy volunteers (76). The flg-/- mice exhibited severe clinical symptoms such as increased ear thickness and elevated IL-6 level (77). IL-6 production in adipose tissue is two to three times higher than that in subcutaneous tissue among obese individuals. IL-6 may stimulate JAK1 activation and phosphorylation, which in turn activates STAT1, STAT3, and STAT6 signaling pathways (78). IL-6 activates the STAT3 pathway of local macrophage to promote IL4Ra expression, sensitizing them to IL-4 signaling and promoting an anti-inflammatory gene expression pattern (79). Additionally, adipocytes release IL-6, promoting differentiation of Th cells, either directly or indirectly, thereby stimulating antibody production (80). IL-6/STAT3, together with TGF-β or IL-1β and IL-23 causes the differentiation of Th17 cells, which play a pro-inflammatory role. IL-6 promotes an HFD-induced increase in FFA and leptin release from adipocytes (81). Research also reports that leptin stimulates macrophages to generate IL-6 via synergistic interaction with LPS (82).

### 5.3 IL-4 and IL-13

IL-4 and IL-13 negatively influence the skin barrier in AD. They downregulate FLG expression, destroy the skin structure, reduce its capacity to resist pathogens and allergens as well as weaken the capacity to maintain adequate moisture (83). IL-4 and IL-13 increase the proliferation of keratinocytes, reduce their differentiation, and prevent their full maturation (84). IL-13 also downregulates the expression of skin barrier proteins and lipids of keratinocytes by mediating MMP-9 expression. Recent studies have shown that IL-4 and IL-13 weaken skin resistance to pathogens by reducing antimicrobial peptides, hence rendering skin more susceptible to infectious organisms (85). IL-13 is also implicated in the upregulation of collagen degradation and fibrosis mediated by MMP-13, causing fibrosis, dermis thickening, and the typical lichenified lesions in AD (86). IL-4 and IL-13 also promote neurogenic itch by directly acting on pruriceptive sensory neurons (87). Th2 pathway activation mediates type 2 inflammation in AD. Th2 cells may release vital inflammatory cytokines including IL-4, IL-13, IL-5, and IL-9, recruit eosinophils and mast cells, as well as stimulate B cell activity (82). Through their interactions with Th2 cells, M2 macrophages in white adipose tissue activate the release of Th2 cytokines, including IL-4 and IL-13 (88). A few studies have shown that obesity exerts IL-4/IL-13-associated inflammatory responses. Elsewhere, the typical Th2-dominant inflammation of AD progressed to more severe Th17-driven inflammation in obese mice. Biologic treatments inhibiting cytokines IL-4 and IL-13 treatment protects lean mice from developing AD but not obese mice (89). IL-6 secreted by inflammatory-stimulated adipocytes activates macrophages STAT3 and upregulates IL4ra expression, and increases the sensitivity of macrophages to IL-4 activation (79). Hypercholesterolemia induces strong Th2 responses to an exogenous antigen characterized by an increased induction of IL-4 and IL-10 (90).

### 5.4 Adipokines

Expanded fat cells themselves produce various signaling molecules through a process called autocrine signaling. These mediators have both immune system regulatory functions as well as metabolic functions, and are collectively called adipokines. For example, chemokines promote the infiltration of macrophages into white adipose tissue (WAT), while calcium-binding proteins enhance the adhesion of circulating monocytes and their recruitment as macrophages. Adipokines include pro-inflammatory ones like leptin, as well as anti-inflammatory cytokines like adiponectin. However, adiponectin levels are suppressed in both acute and chronic obesity.

### 5.5 Leptin

Leptin is a 16-kDa monomeric non-glycosylated protein primarily secreted by adipocytes. Circulating leptin levels are directly proportional to body fat mass (91). Leptin activates CD4+T lymphocytes toward Th1 phenotype and inhibits infiltration of Tregs into adipose tissue, altered immune tolerance, and inflammatory effects (92). Leptin promotes the release of IL-2 and INF-γ. IL-2 acts on IL-2R, stimulating JAK1 and JAK3 phosphorylation, further activating as well as phosphorylating STAT1, STAT3, STAT5, and STAT6. The INF-γ signaling pathway involves JAK1 and JAK2 phosphorylation, hence activating and phosphorylating STAT1 (93). The binding of leptin to LEP-R also activates the phosphoinositide 3-kinase (PI3K) and mitogen-activated protein kinase/extracellular signal-regulated kinase (MAPK/ERK) signaling cascades, as well as the JAK2-STAT signaling cascade (57). Additionally, chronic treatment of obesity with leptin promotes preadipocyte differentiation and secretion of pro-inflammatory cytokine TNF-α (94). Leptin increases TNF and IL-6 production by monocytes as well as stimulates CCL3, CCL4, and CCL5 production by macrophages via JAK2–sTAT3 pathway activation (95). Leptin also induces TNF-α expression via the JNK pathway in macrophages (96). A positive feedback mechanism is established when leptin stimulates inflammatory mediator production, including IL-6 and TNF-α from the adipose tissue. TNF-α and IL-6 promote the expression and release of leptin.

### 5.6 Adiponectin

One study observation revealed a potential relationship between adiponectin and AD, i.e., obese children with asthma had higher leptin levels and lower adiponectin levels in serum than non-obese children with asthma (97). In BMDC cells, adiponectin induces DC maturation and activation, accelerating naive T cells polarized into Th1 and Th17 cells. High-fat diet-fed upregulates IFN-γ expression in adipocytes and IL-17 in CD4 T cells (98). Additional experiments have shown that adiponectin derived from adipocytes reduces T lymphocytes, thus producing IFN-γ and IL-17 (99). Adiponectin-deficient mice had higher TNFα levels in the blood. Adiponectin abrogated LPS-stimulated production of TNF-α in macrophages and suppressed TLR-mediated NF-κB activation in mouse macrophages (100). In this context, adiponectin may exert anti-inflammatory mechanisms in AD. However, additional studies are necessary to comprehensively understand this mechanism.

### 5.7 Resistin

Resistin, named as a result of inducing insulin resistance, is a 12.5-kDa cysteine-rich peptide, produced by macrophages and peripheral monocytes. Nevertheless, the potential role of resistance in AD is unclear. Studies revealed that blood content of resistin increases in AD boys unlike in healthy children (101). Besides, resistin upregulates pro-inflammatory cytokines including TNF-α, IL-1β, IL-6, and IL-12 via NF-kB signaling pathway activation (102). However, other findings contradict previous results. Studies also discovered a decrease of resistin in AD patients, with an inverse correlation between blood resistin quantity and SCORAD score (103, 104). Notably, resistin weakens the atopic immune response by suppressing dendritic cell function (92). Thus, lower levels of resistin are thought to be associated with increased severity of AD symptoms in adults. Nonetheless, additional studies are necessary to define and validate the role of resistin in AD.

### 5.8 Fatty acid binding protein

Fatty acid binding protein (FABP) regulates fatty acid uptake, transport, and metabolism. Epidermal-FABP (FABP5), an extensively studied member of the FABP family, was has been reported to be positively correlated with adiposity, glucose metabolism, and lipolysis parameters and linked to the development of AD (105, 106). Mass spectrometry of AD skin revealed that FABP5 is highly expressed in both acute and chronic AD lesions (107). Elsewhere, it was demonstrated that FABP5 promoted TNF-α-induced NF-κB signaling by forming a complex with valosin-containing protein (VCP) in keratinocytes (108). Another study provided evidence supporting a Th17-related mechanism in AD, involving FABP5. Knockdown of FABP5 in HaCaT cells resulted in a significant reduction of the expression of Th17-inducing cytokines, including IL-6 and TGF-β (109). In HFD-induced obese mice models, FABP5 expression in skin macrophages promoted saturated FA-induced IL-1β production and instigated chronic inflammatory skin lesions (110). Indeed, adipose-FABP (FABP4) and FABP5 regulate different signaling pathways in macrophages. Although FABP5 expression activates the STAT1/2/IFNβ, LTA4/LTB4, RAR/CD11c, or NLRP3/IL-1β pathways, FABP4 mainly stimulates NFκB/IL-6, COX2/PGE2, CER/cell death, or LXR/SCD1 in macrophages (111).

### 5.9 Zinc-α2-glycoprotein

Zinc-α2-glycoprotein (ZAG), a 43-kDa protein produced by adipocytes and keratinocytes in the skin plays a role in lipolysis in adipocytes (112). In sera and skin of AD patients, it was reported that the expression of ZAG was consistently reduced. ZAG regulated FLG and TSLP expression in normal human epidermal keratinocytes (NHEKs) and repaired abnormalities in the skin barrier under AD conditions (113). Furthermore, topical ZAG treatment decreased levels of Il-4, Il-17a, Ifng and levels of serum total IgE, and restored ADAM17 and Notch1 signaling in AD-induced mice (113).

### 5.10 Visfatin

Visfatin, a novel adipocytokine, has been linked to chronic inflammatory diseases. A study suggested a connection between visfatin and both adult-onset AD and classical AD in adults during the chronic phase of the disease. The study also found a significant correlation between visfatin levels and eosinophil counts in AD patients (114). Visfatin induced pro-inflammatory effects by dose-dependently up-regulating IL-1β, IL-1Ra, IL-6, IL-10, and TNF-μ in human monocytes (115). Moreover, it stimulated the production of chemokines such as CXCL8, CXCL10, and CCL20 in human keratinocytes (116). However, the serum concentration of visfatin in AD children was significantly reduced compared to that of healthy subjects (101), which differs from finding of other studies. This is likely due to differences in the underlying mechanism of child AD and adult AD. Some studies have shown that patients with adult-onset AD have significantly higher serum visfatin levels than those who had developed the skin lesions in childhood (113).

### 5.11 Lipocalin-2

Lipocalin-2 (LCN2) is associated with various obesity-related disorders, such as Type 2 diabetes and non-alcoholic fatty liver disease (117). It circulates in the body in two main forms: a single molecule (25 kDa monomer) and a double molecule (46 kDa homodimer). Notably, LCN2 can block the breakdown of MMP-9, an enzyme involved in tissue remodeling (118). Interestingly, research suggests LCN2 may also play a role in AD. A study using a mouse model of AD found upregulated levels of LCN2 in spinal astrocytes, which are cells that support nerve function in the spinal cord. This finding suggests a potential link between LCN2 and itch sensation at the spinal level in AD (119). Furthermore, the LCN2 gene has binding sites for several inflammatory signaling molecules, including NF-κB, STAT1, STAT3, and C/EBP (120).

## 6 PPAR-γ and AD

Peroxisome proliferator-activated receptor-gamma (PPAR-γ) is a critical transcription factor involved in adipogenesis. PPAR-γ plays an important in regulating adipocyte differentiation and lipid metabolism, hence providing positive feedback regulation of adipogenesis. Accumulating evidence indicates that PPAR-γ regulates type 2 immune response (121).

PPAR-γ agonists reduce neutrophil MPO activity in response to LPS and induce neutrophil apoptosis in a dose-dependent manner (122). PPAR-γ activation also impairs the functional capacity of eosinophils, hence reducing antibody-dependent cellular cytotoxicity (ADCC), CD69 expression induced by IL-5, and release of eosinophil-derived neurotoxin (EDN) (123). Mast cell activation mediated by IgE plays a significant role in AD, which is also suppressed by activated PPAR-γ (124). PPAR-γ is a key factor mediating M2 phenotype associated with type 2 cytokine activation (such as IL-4 and IL-13). PPAR-γ prevents the uptake of lipids of ILC2s, IL-33 signaling activation, and reduces the severity of airway inflammation (125). Mice treated with PPAR-γ agonists showed reduced levels of cytokines IL-4, IL-5, and IL-13 (126), whereas mice without PPAR-γ mice revealed enhanced release of epithelial-derived alarm proteins including IL-25, IL-33, and TSLP as well as NF-κB activation (127). However, the effect of PPAR-γ deletion on IL-4 expression was unclear (128). Activated PPAR-γ reduces the expression of pro-inflammatory cytokines including TNF-α, IFN-γ, and IL-2 as well as promotes the activity of suppressive Treg cells (129).

Experimental evidence indicates that PPAR-γ in epithelial cell Treg cells is activated by TCR signaling in an IRF4-dependent manner. PPAR-γ binds to IRF4 and modulates IL-33 receptor (IL-33R) expression on Treg cells (130). The role of PPAR-γ in other TH cell subsets has also been proposed. IL-9 expression in the skin positively correlates with the severity of chronic atopic dermatitis (cAD) and acute contact dermatitis reactions (aACD) (131). TH9 cells can be perceived as a highly differentiated subset of TH2 cells that can simultaneously promote the levels of IL-5, IL-13, and IL-9 (128). IL-9 is preferentially downregulated among the TH2 cytokines upon inhibition of PPAR-γ expression (132). The relationship between TH9 immune response and AD is poorly understood. However, cytokine secretion including IL-5 and IL-13 by TH9 cells implies a potential relationship with AD.

## 7 Conclusion

**Figure 1:**
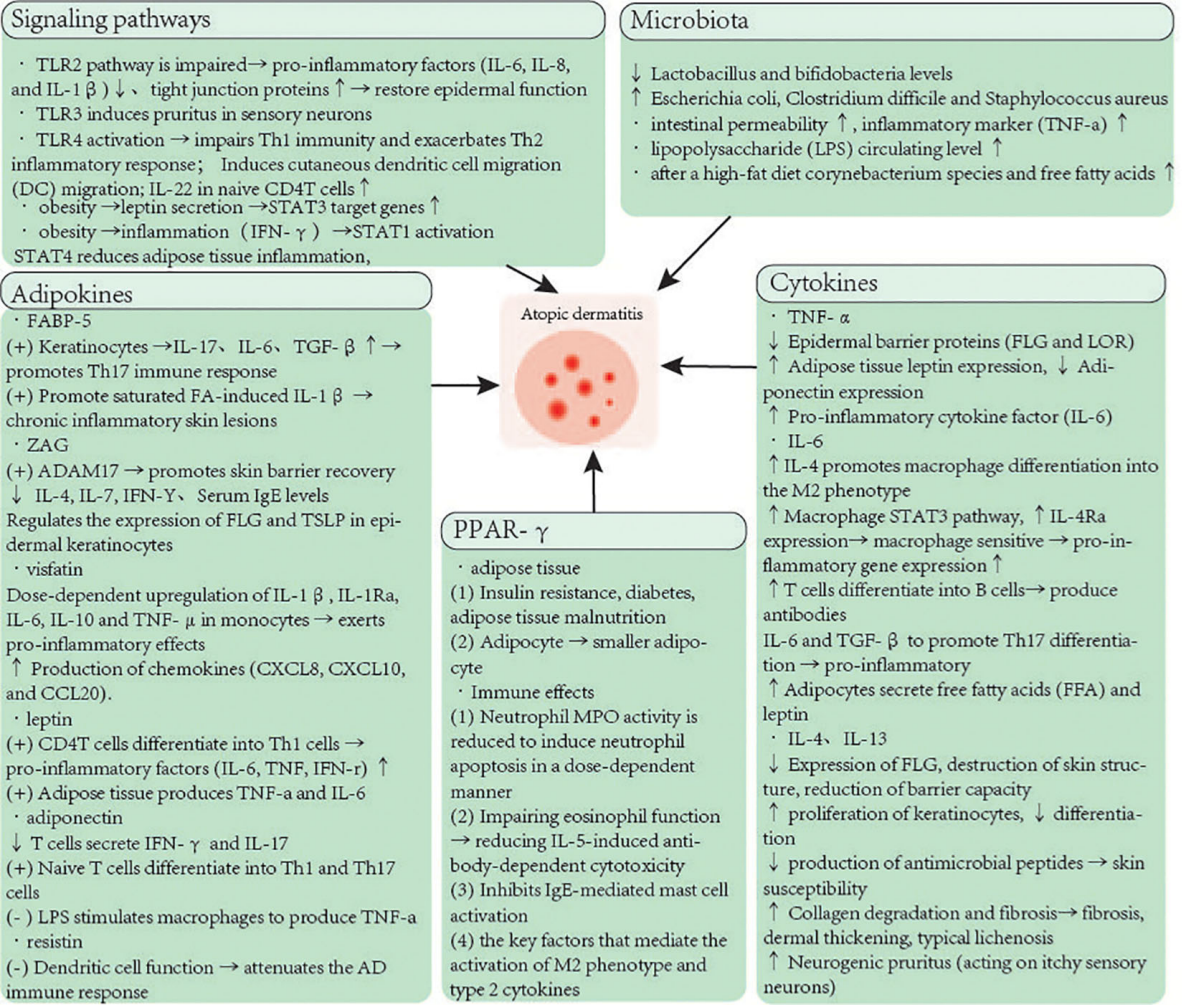
The molecular mechanisms of obesity predisposes to AD.

Current clinical research and scientific studies suggest that overweight or obesity are considered primary factors leading to the pathogenesis of inflammatory skin diseases. Our review covers multiple important aspects. Under the influence of initial inflammatory factors, obese individuals experience a complex interplay of various pro-inflammatory and anti-inflammatory signals within their bodies. Inflamed adipocytes locally and systemically secrete pro-inflammatory cytokines, a process that damages both adipose tissue itself and skin functions. Another key factor in obesity involves the gut microbiota, which plays a role in energy homeostasis, circulatory system, and immune response. The increase in skin lipids following obesity can lead to dysbiosis of the microbial ecosystem, resulting in colonization by lipophilic bacteria. Concurrently, obesity also alters the pathological changes of inflammatory diseases, shifting the classical Th2-type immune response to a more severe Th17-type immune response through the action of adipokines, thereby affecting keratinocyte differentiation, epithelial barrier permeability, and antimicrobial peptide production. Simultaneously, maintaining balance in the gut microbiota and probiotics play a complex role in preventing atopic dermatitis (AD), including inhibition of inflammation, alteration of microbial diversity, and enhancement of skin barrier function. These contents provide more thinking and inspiration about AD, and will have a positive impact on the field of AD (**Figure 1**).

More data is needed to understand how weight and BMI affect the effectiveness of AD therapy. Reducing weight and managing obesity might decrease inflammatory mediators and cytokines from adipose tissue, thereby improving the inflammatory state and alleviating AD symptoms. As such, besides targeting skin lesions in the treatment of AD, the management and intervention of obesity should also be emphasized to comprehensively contain disease progression.
