# Correction: Molecular mechanisms of obesity predisposes to atopic dermatitis

**DOI:** 10.3389/fimmu.2026.1850518

**Published:** 2026-04-20

**Authors:** Dajin Shang, Shengnan Zhao

**Affiliations:** 1 School of China Medical University, Shenyang, Liaoning, China; 2 Department of Dermatology, The First Hospital of China Medical University, Shenyang, China

**Keywords:** atopic dermatitis, obesity, immune, adipokines, cytokines

Author Shengnan Zhao was erroneously assigned to affiliation “School of China Medical University, Shenyang, Liaoning, China.” This affiliation has now been removed for author Shengnan Zhao.

The original version of this article has been updated.

